# *De Novo* CNV Formation in Mouse Embryonic Stem Cells Occurs in the Absence of Xrcc4-Dependent Nonhomologous End Joining

**DOI:** 10.1371/journal.pgen.1002981

**Published:** 2012-09-20

**Authors:** Martin F. Arlt, Sountharia Rajendran, Shanda R. Birkeland, Thomas E. Wilson, Thomas W. Glover

**Affiliations:** 1 Department of Human Genetics, University of Michigan, Ann Arbor, Michigan, United States of America; 2 Department of Pathology, University of Michigan, Ann Arbor, Michigan, United States of America; Memorial Sloan-Kettering Cancer Center, United States of America

## Abstract

Spontaneous copy number variant (CNV) mutations are an important factor in genomic structural variation, genomic disorders, and cancer. A major class of CNVs, termed nonrecurrent CNVs, is thought to arise by nonhomologous DNA repair mechanisms due to the presence of short microhomologies, blunt ends, or short insertions at junctions of normal and *de novo* pathogenic CNVs, features recapitulated in experimental systems in which CNVs are induced by exogenous replication stress. To test whether the canonical nonhomologous end joining (NHEJ) pathway of double-strand break (DSB) repair is involved in the formation of this class of CNVs, chromosome integrity was monitored in NHEJ–deficient *Xrcc4* ^−/−^ mouse embryonic stem (ES) cells following treatment with low doses of aphidicolin, a DNA replicative polymerase inhibitor. Mouse ES cells exhibited replication stress-induced CNV formation in the same manner as human fibroblasts, including the existence of syntenic hotspot regions, such as in the *Auts2* and *Wwox* loci. The frequency and location of spontaneous and aphidicolin-induced CNV formation were not altered by loss of Xrcc4, as would be expected if canonical NHEJ were the predominant pathway of CNV formation. Moreover, *de novo* CNV junctions displayed a typical pattern of microhomology and blunt end use that did not change in the absence of Xrcc4. A number of complex CNVs were detected in both wild-type and *Xrcc4* ^−/−^ cells, including an example of a catastrophic, chromothripsis event. These results establish that nonrecurrent CNVs can be, and frequently are, formed by mechanisms other than Xrcc4-dependent NHEJ.

## Author Summary

Copy number variants (CNVs) are a major factor in genetic variation and are a common and important class of mutation in genomic disorders, yet there is limited understanding of how many CNVs arise and the risk factors involved. One DNA damage response pathway implicated in CNV formation is nonhomologous end joining (NHEJ), which repairs broken DNA ends by Xrcc4-dependent direct ligation. We examined the effects of loss of Xrcc4 and NHEJ on CNV formation following replication stress in mouse cells. Cells lacking NHEJ displayed unaltered CNV frequencies, locations, and breakpoint structures compared to normal cells. These results establish that CNV mutations in a cell model system, and likely *in vivo*, arise by a mutagenic mechanism other than canonical NHEJ, a pattern similar to that reported for model translocation events. Potential roles of alternative end joining and template switching are discussed.

## Introduction

The importance of genomic copy number variants (CNVs), defined as submicroscopic deletions or duplications ranging in size from 50 bp to over a megabase [1], has become better understood in recent years. Normal polymorphic CNVs are a major contributor to human genomic variation and phenotypic diversity [2], [3], [4], [5], [6], while spontaneous CNVs are a very important and frequent cause of genetic and developmental disorders, including intellectual disability, neuropsychiatric disorders, and structural birth defects [7], [8], [9], [10], [11], [12], [13], [14]. Their frequency further suggests a high *de novo* mutation rate, with estimates between 0.01 and 0.05 per meiosis [6], [15], [16], [17]. In addition, CNVs are likely to be but one manifestation of the same mutagenic forces that create many classes of chromosomal structural variants, including copy-number neutral inversions and translocations [18], [19], [20].

While there is growing appreciation for their importance, less is understood about how many CNVs are formed. Recurrent CNVs arise during meiosis by nonallelic homologous recombination (NAHR) in regions flanked by large segmental duplications [21]. In contrast, nonrecurrent CNVs are distributed throughout the genome in regions lacking such homologous sequences. These CNVs have breakpoint junctions that are characterized by blunt ends, microhomologies, and small insertions, suggesting the involvement of a nonhomologous repair mechanism in their formation [22], [23], [24], [25]. A number of different DNA repair mechanisms have been suggested to account for nonhomologous junctions, principally nonhomologous end-joining (NHEJ), alternative end-joining (alt-EJ), and forms of replication template switching [26].

Canonical NHEJ, along with homologous recombination (HR), is one of the two major mechanisms used to repair DNA double-strand breaks (DSBs) in eukaryotic cells. NHEJ directly joins two DSB ends without using extensive sequence homology to guide repair through the action of a well-defined set of proteins, including the Xrcc4-ligase IV complex, which is dedicated to and essential for this pathway [27]. The junctions formed are typically characterized by blunt ends or short microhomologies and can include insertions of a few nucleotides [26], [28]. NHEJ can ligate distant DSBs to form deletions [29]. Consistently, NHEJ has been implicated in the formation of deletion CNVs [22], [25], [30], [31], [32]. In a two-step mechanism combined with HR, NHEJ has also been suggested to be involved in the formation of duplications [23], [30], [33].

Xrcc4-ligase IV-independent forms of DSB end joining also exist, variably called alt-EJ or microhomology-mediated end joining. Alt-EJ is ordinarily less efficient than and/or suppressed by NHEJ such that its activity is often revealed principally in the absence of NHEJ proteins. For example, in the absence of Xrcc4-ligase IV, alt-EJ becomes important in class-switch recombination [34] and executes an increased frequency of translocations in a two-DSB model system [35]. The alt-EJ mechanism(s) are much less well defined than NHEJ, but repair events are typically characterized by longer stretches of microhomology at junctions, thought to arise mainly through annealing of single strands exposed by DSB resection [28], [36], [37]. Accordingly, alt-EJ is strongly mutagenic.

In contrast to end joining mechanisms, which obligatorily proceed through DSB intermediates and could occur throughout the cell cycle, mechanisms based on replication template switching have also been proposed to explain the presence of microhomologies at CNV junctions. Lee et al. [23] proposed the Fork Stalling and Template Switching (FoSTeS) model in which replicating DNA strands switch between forks. A revision of this model, termed microhomology-mediated break-induced replication (MMBIR) [37], invokes one single-ended DSB intermediate at a collapsed replication fork at which a liberated DNA strand makes the template switch into a distant genomic site. These models are supported by complex CNVs in humans and mice that can be explained by multiple template switching events [19], [38], [39], [40], as well as by deletions and duplications occurring independently of breakage fusion bridge cycles near fused telomeres in *C. elegans* [41]. However, as with alt-EJ, mammalian proteins involved in this process are not well defined.

We previously reported an experimental approach for inducing *de novo* CNVs that closely mimic the nonrecurrent class of human CNVs [42], [43], [44]. In this approach, mild replication stress resulting from low doses of the replication inhibitors aphidicolin and hydroxyurea potently induces formation of *de novo* CNVs that resemble nonrecurrent CNVs *in vivo* in size and structure. In particular, both human nonrecurrent CNVs and those induced in our experimental system have breakpoint junctions primarily characterized by microhomologies, blunt ends, or small insertions. These observations led to the hypothesis that NHEJ participates in the formation of nonrecurrent CNVs, although their induction by replication stress and the occurrence of complex events also raised the possibility of template switching mechanisms [23], [37], [42], [43].

For both experimentally-induced CNVs and those occurring in humans *in vivo*, the proposed mechanisms have only been inferred from junction sequences; direct experimental tests have been lacking. Because of the strong functional implications of the potential alternative mechanisms of nonrecurrent CNV formation, we sought to definitively explore the role of the well-defined, canonical NHEJ pathway. We report studies of CNV formation using *Xrcc4* ^−/−^ mouse embryonic stem (ES) cells and the DNA polymerase inhibitor aphidicolin (APH). Xrcc4 is an essential component of DNA ligase IV that is absolutely required for NHEJ [27]. We demonstrate that APH induces *de novo* CNVs in mouse ES cells as it does in human fibroblasts, but that there is no difference in the frequency or structure of spontaneous or induced CNVs between wild-type and *Xrcc4^−/−^* cells. CNV breakpoint junctions were characterized by blunt ends and microhomologies regardless of genotype, with no observed shift in microhomology lengths. We conclude that replication-associated CNVs in mouse ES cells are created through mechanism(s) other than canonical NHEJ and discuss the potential roles of alt-EJ and template switching in the context of both simple and complex CNVs observed in the presence and absence of Xrcc4.

## Results

### Xrcc4 deficiency does not reduce the frequency of APH-induced CNVs

To document the validity of our cell model, PCR was used to demonstrate the presence of a homozygous inactivating *Xrcc4* deletion mutation in the *Xrcc4^−/−^* mouse ES cells used in these studies. Supporting this, these cells also demonstrated a large decrease in survival after exposure to ionizing radiation (Figure S1), consistent with NHEJ deficiency. Prior to the experiments, parental cells of each genotype were expanded from a single clone to minimize the number of potentially mosaic CNVs in the starting cell population. To induce replication stress, wild-type and *Xrcc4^−/−^* mouse ES cells were cultured in the presence of 0–0.6 µM APH for 72 hours prior to plating for isolation of clonal cell populations. This mild dose of APH does not block the cell cycle, but instead allows replication to proceed at a reduced rate. Individual clones were expanded and subjected to CNV analysis using Nimblegen 3x720K aCGH arrays (Figure S2). *De novo* CNVs were defined as a segmental gain or loss detected in a clone when using the parental cell population as a reference.

**Figure 1:**
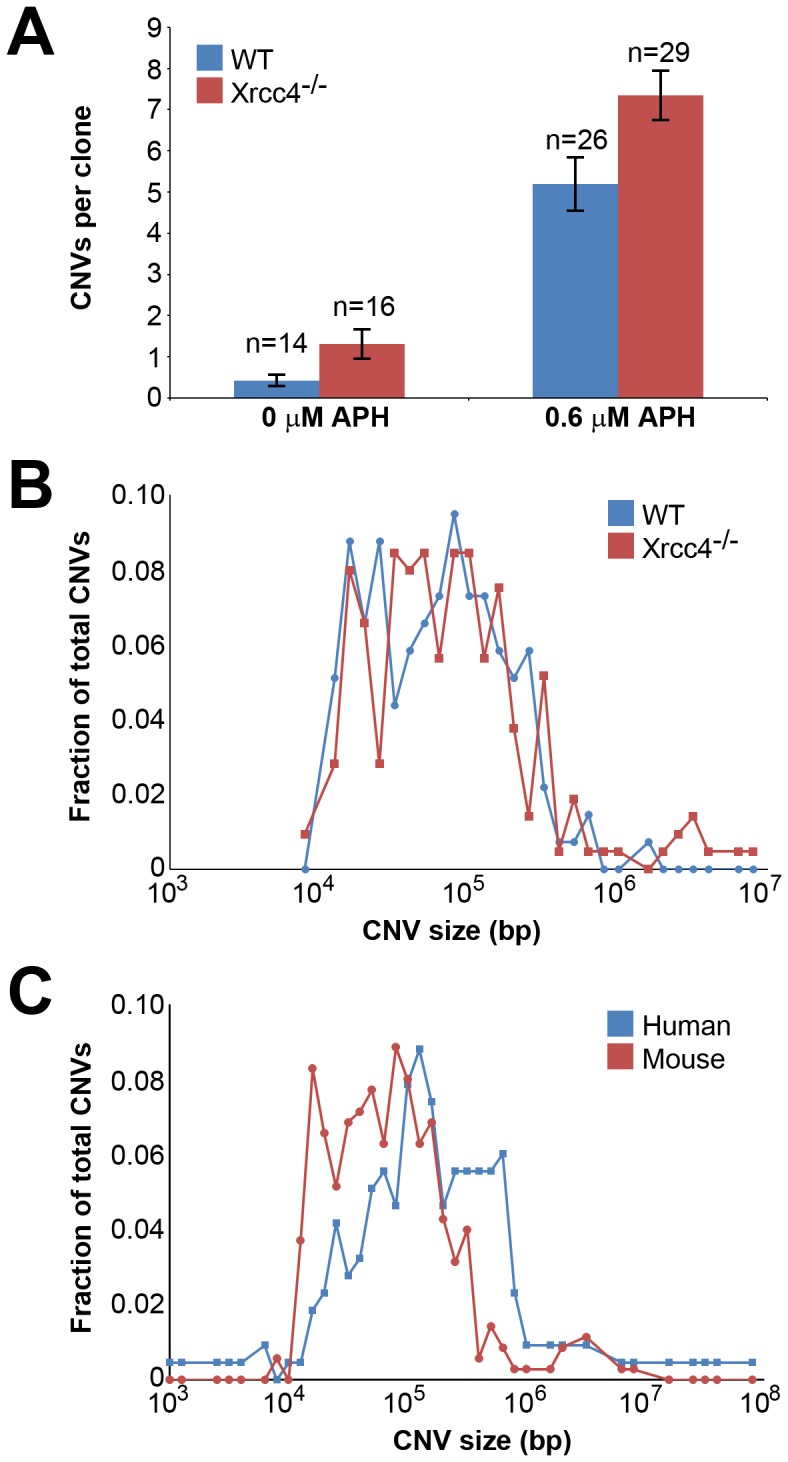
Replication stress induces CNVs in mouse ES cells. (A) Incidence of spontaneous and induced CNVs in wild-type and *Xrcc4^−/−^* cells treated with 0.0–0.6 µM APH for 72 hours. A total of 85 independent clones of untreated and treated, wild-type and *Xrcc4^−/−^* cells were analyzed. Error bars indicate standard error. (B) Size distribution of *de novo* CNVs in wild-type (blue) and *Xrcc4^−/−^* cells (red). (C) Size distribution of *de novo* CNVs in human fibroblasts (blue) and mouse ES cells (red).

A total of 85 independent clones from untreated or APH-treated wild-type and *Xrcc4^−/−^* cells were analyzed in three independent experiments. In wild-type cells, *de novo* CNVs were found in untreated and APH-treated clones at a frequency of 0.43 and 5.19 CNVs per clone, respectively (p<10^−14^) (Figure 1A), demonstrating that, just as in previous studies with human fibroblasts, *de novo* CNVs in mouse ES cells can arise spontaneously during culture but that their frequency is significantly increased following replication stress.

In *Xrcc4^−/−^* cells, *de novo* CNVs were identified in untreated and APH-treated clones at a frequency of 1.31 and 7.34 *de novo* CNVs per clone, respectively (p<10^−14^) (Figure 1A). When all experiments are considered together as in Figure 1A, there appears to be a slight increase in CNV induction in *Xrcc4^−/−^* cells compared to wild-type cells. However, this effect was only seen in one experiment in which APH-treated, wild-type cells had an unusually low CNV frequency. It was not recapitulated in the two subsequent experiments (Figure S3). Thus, there was no consistent effect of Xrcc4 deficiency on the frequency of spontaneous or APH-induced *de novo* CNV formation in ES cells. Most importantly, *Xrcc4* deficiency did not reduce the frequency of CNVs, as might be expected if NHEJ were the predominant pathway of CNV formation.

### *De novo* CNV sizes in wild-type and *Xrcc4^−/−^* cells

Because NHEJ deficiency might affect CNV structure independently of frequency, we compared numerous features of *de novo* CNVs in wild-type and *Xrcc4^−/−^* cells. While CNVs consisted of a mix of both deletions and duplications, there was a clear overrepresentation of deletions in both wild-type and *Xrcc4^−/−^* cells. 130 of 143 (90.9%) CNVs from wild-type cells and 195/234 (83.3%) CNVs from *Xrcc4^−/−^* cells were of the deletion type. The abundance of deletions compared to duplications is consistent with results seen in normal human fibroblasts after replication stress, in which 65–82% of CNVs were deletions [42], [43], and in humans *in vivo* [6].

There was no difference in overall *de novo* CNV size between wild-type and *Xrcc4^−/−^* cells (Figure 1B). *De novo* CNVs in wild-type cells were generally large, with a median size of 59 kb (11.6 kb to 1.4 Mb). These sizes are similar to *de novo* CNVs seen in *Xrcc4^−/−^* cells, which had a median size of 63 kb (7.7 kb to 26.2 Mb). We did note that CNVs arising in mouse ES cells (median = 62 kb) were 2.2-fold smaller than *de novo* CNVs seen in similar experiments with human fibroblasts (median = 138 kb) [42] (Figure 1C).

### Locations of *de novo* CNVs in wild-type and *Xrcc4^−/−^* cells

**Figure 2:**
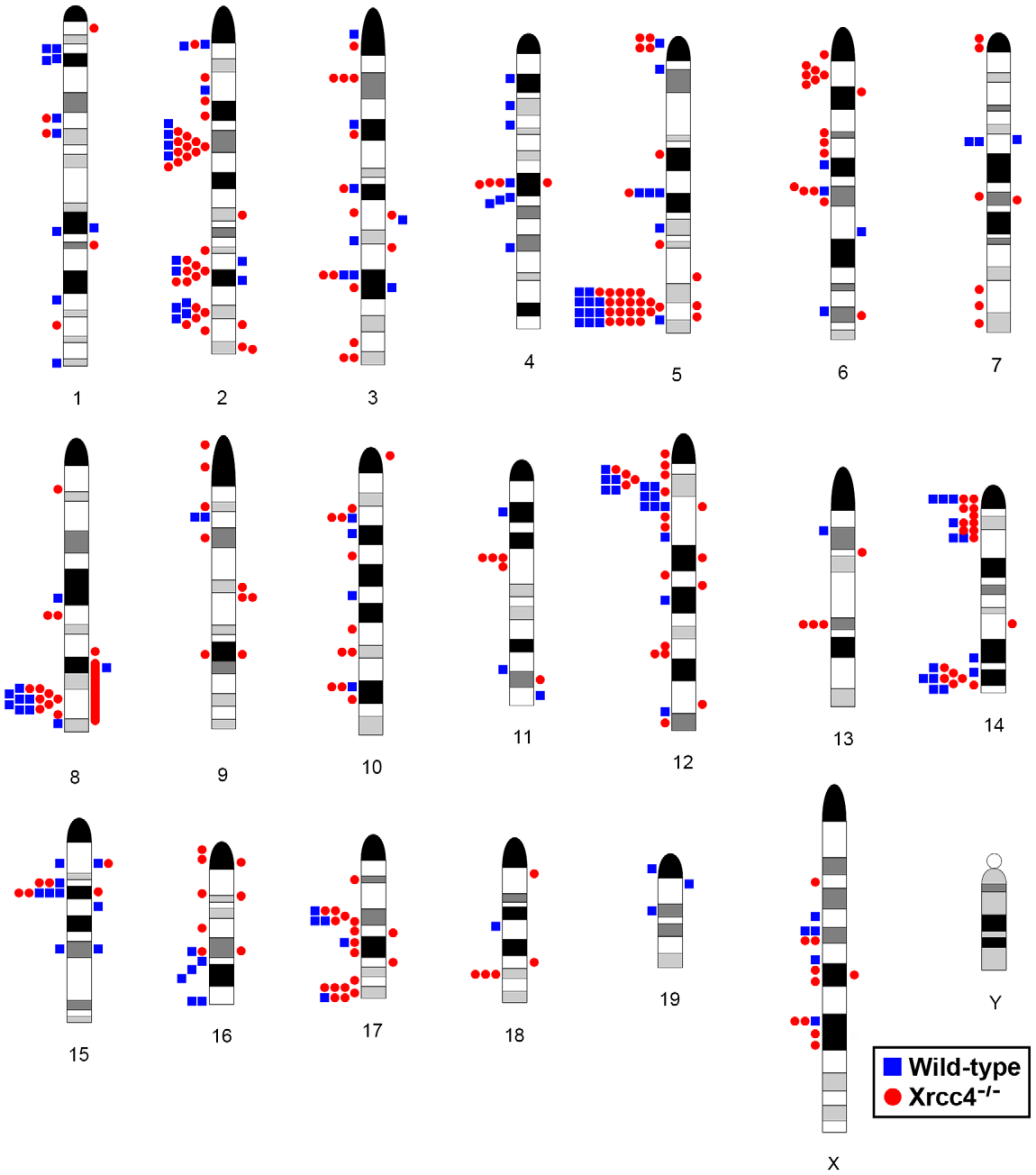
Locations of *de novo* CNVs in the mouse genome. CNVs are mapped onto a mouse chromosome ideogram. Blue squares indicate *de novo* CNVs in wild-type cells. Red circles indicate *de novo* CNVs in *Xrcc4^−/−^* cells. Symbols to the left of a chromosome represent deletions and symbols to the right represent duplications. Ideograms adapted from www.pathology.washington.edu/research/cytopages/idiograms/mouse (Dept. of Pathology, University of Washington, with permission). Precise coordinates for all *de novo* CNVs are listed in Table S1.

Consistent with previous observations in human fibroblasts, spontaneous and APH-induced CNVs in both wild-type and *Xrcc4^−/−^* mouse ES cells were distributed throughout the genome, with most arising in distinct, nonoverlapping regions (Figure 2). Superimposed on this distribution pattern were hotspots containing five or more different, overlapping CNVs, a number identified as unexpected by simulation modeling (see Materials and Methods). Each CNV within these hotspots had unique boundaries, indicating that each one arose independently, supporting the hypothesis that these regions are especially sensitive to replication stress. A difference in the size distribution of CNVs at hotspots and non-hotspots was observed, with hotspot CNVs being on average 1.9-fold larger than non-hotspot CNVs (median sizes of 89.8 kb and 46.3 kb, respectively). In addition, the abundance of deletions over duplications was more pronounced at hotspots than at non-hotspots. At non-hotspots, 79.5% (194/244) of *de novo* CNVs were of the deletion type, whereas at hotspots, almost all *de novo* CNVs (98.5%; 131/133) were deletions (p<0.0001). Most importantly, there was no apparent difference in the spatial distribution of CNVs between wild-type and *Xrcc4^−/−^* cells, including that hotspots accounted for 35.0% (50/143) and 35.5% (83/234) of all *de novo* CNVs, respectively.

**Figure 3:**
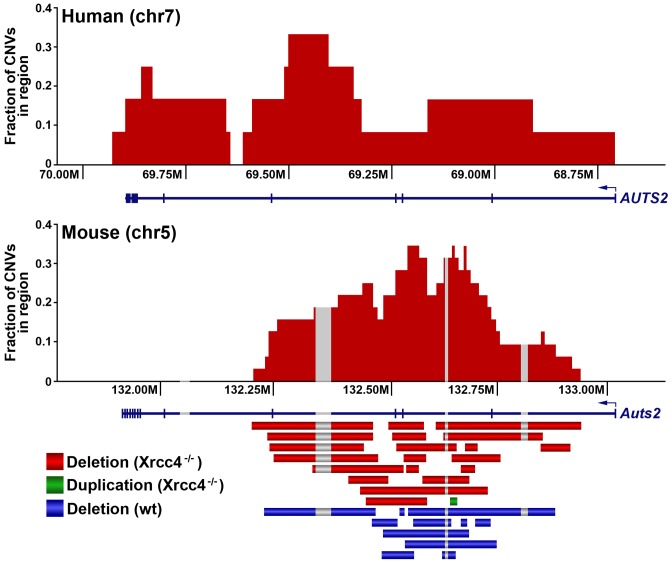
A conserved CNV hotspot in mouse and human cells. A mouse CNV hotspot at 5G2 in *Auts2* corresponds to a previously-described human CNV hotspot at human 7q11.2 in the *AUTS2* gene [42]. The x-axis shows the position along the chromosome, while the y-axis indicates that fraction of hotspot CNVs that crossed a particular 10 kb genomic window. CNVs detected in mouse ES cells are depicted as bars. Gray areas indicate regions of inserted sequences in the human relative to mouse genomes. Although overlapping CNVs were found in these regions, all had distinct breakpoints.

Notably, several ES cell hotspots were found in the syntenic regions corresponding to hotspots seen in human fibroblasts (Table 1, Figure 3, Figure S4). The mouse hotspot with the most frequent occurrence of CNVs was in the *Auts2* gene at chromosome 5G2. One or more CNVs in *Auts2* were seen in 28/55 APH-treated clones, and accounted for 8.5% of all *de novo* CNVs. In addition, a hotspot was seen in the *Wwox* gene at 8E1, with CNVs found in 12/55 APH-treated clones. Both of these hotspots corresponded to hotspots seen in human fibroblasts (Figure 3, Table 1). However, the most frequently observed hotspot in human fibroblasts, at 3q13.31, was not a hotspot in mouse ES cells. In fact, most (11/13) of the hotspots in mouse ES cells were not observed in human fibroblasts (Table 1). In addition, two hotspots corresponded to the common fragile sites Fra8E1 (FRA16D) and Fra14A2 (FRA3B), while others mapped to regions syntenic to human fragile sites. These results suggest that while there is some conservation in replication stress-induced CNV hotspots, differences are also seen due to cell type or species variation.

**Table 1:** *De novo* CNV hotspots in mouse ES cells.

Chr	Hotspot Region Start	Hotspot Region End	Size	Associated genes	Number of CNVs at hotspot (n = 377)	Hotspot CNV frequency per APH-treated clone (n = −55)	Mouse fragile site	Human Syntenic Region	Human CNV hotspot?^a^	Human fragile site
2C1.3	67,227,959	67,873,163	645,204	Xirp2	15 (4.0%)	27.3%		2q24.3		FRA2T^b^
2F3	140,550,331	141,557,233	1,006,902	Macrod2	9 (2.4%)	16.4%		20p12.1		FRA20B^c^
2H2	161,777,721	162,229,484	451,763	Ptprt	8 (2.1%)	14.5%		20q12		
5A1	4,957,925	5,164,013	206,088	Cdk14	5 (1.3%)	9.1%		7q21.13		
5G2	132,196,231	132,935,046	738,815	Auts2	32 (8.5%)	58.2%		7q11.22	+	FRA7J^d^^,^ c
6A2	21,333,255	21,447,908	114,653	Kcnd2	6 (1.6%)	10.9%		7q31.31		
8E1	117,160,242	117,608,147	447,905	Wwox	14 (3.7%)	25.5%	Fra8E1	16q23.1	+	FRA16D
12A3	35,023,371	35,455,164	431,793	Hdac9	10 (2.7%)	18.2%		7p21.1		
12B1	38,642,601	39,183,139	540,538	Dgkb	5 (1.3%)	9.1%		7p21.2		
14A1	10,761,778	10,964,749	202,971	Fhit	5 (1.3%)	9.1%	Fra14A2	3p14.2		FRA3B
14E4	117,568,872	117,958,464	389,592	Gpc6	10 (2.7%)	18.2%		13q31.3		Unnamed^d^
17C	50,995,219	51,146,542	151,323	Tbc1d5	7 (1.9%)	12.8%		3p24.3		FRA3A^c^
17E5	90,801,260	91,269,143	467,883	Nrxn1	7 (1.9%)	12.8%		2p16.3		FRA2D^c^

(a) Arlt et al., 2009; Arlt et al., 2011.

(b) Mrasek et al., 2010.

(c) Fragile site not molecularly characterized, mapped to region cytogenetically.

(d) Human band corresponds to APH-induced fragile site in fibroblasts (Le Tallec et al, 2011).

### CNV breakpoints in *Xrcc4^−/−^* cells show blunt ends, short microhomologies, and small insertions

**Figure 4:**
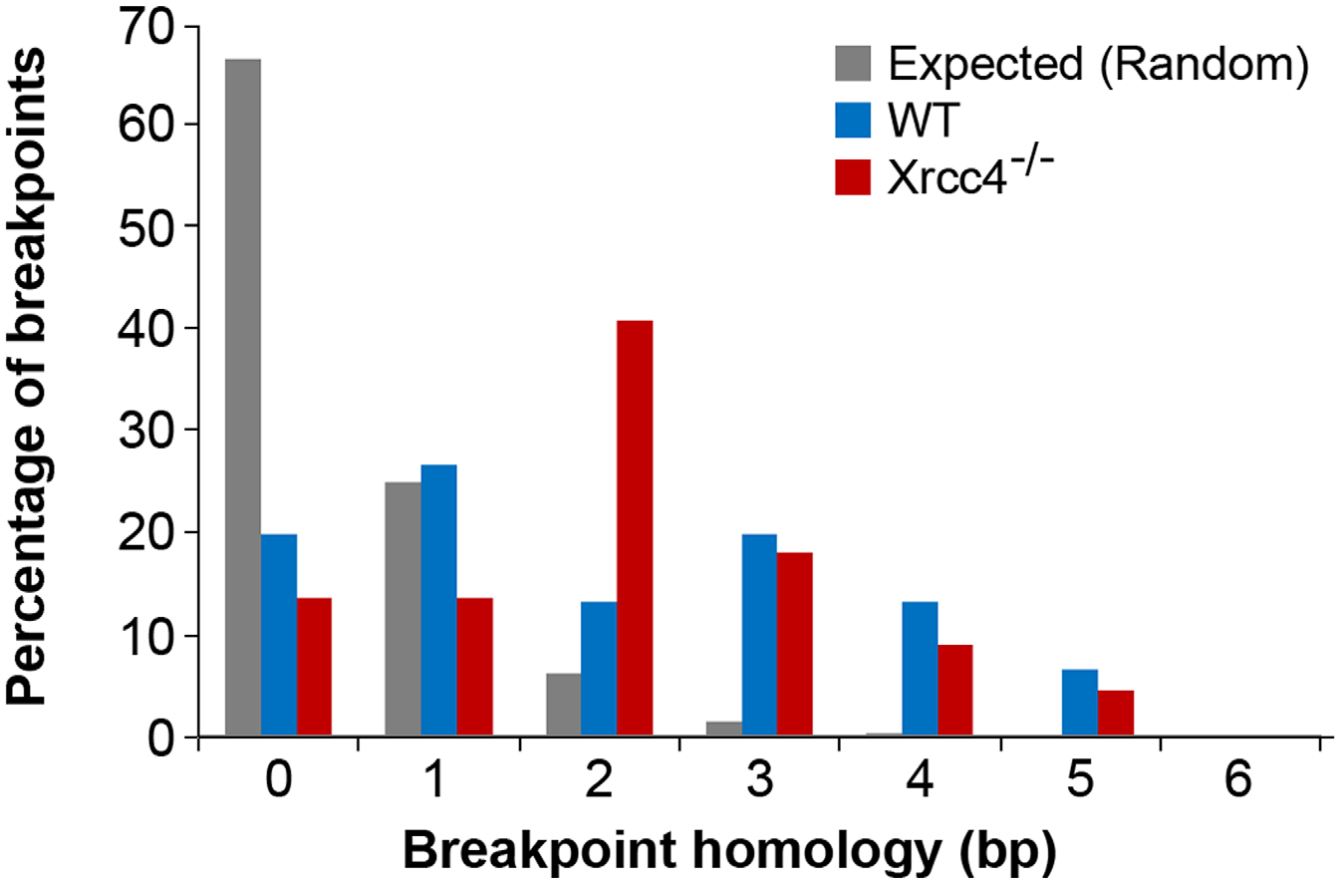
Comparison of observed *de novo* CNV breakpoint junction sequence homology in wild-type and *Xrcc4^−/−^* cells. Histogram showing CNV breakpoint homology in wild-type (blue) and *Xrcc4^−/−^* cells (red), compared to the expected distribution if microhomology usage was random (gray).

In the absence of canonical NHEJ, the pattern of breakpoint junction sequences provides the most precise structural signature for revealing altered utilization of different end joining repair mechanisms [28], [36], [37]. To examine this, we sequenced 24 CNV breakpoint junctions from *Xrcc4^−/−^* cells and 17 from wild-type cells (Table 2)(Figure 4). All of the junctions from both wild-type and *Xrcc4^−/−^* cells were characterized by 0–5 bp of homology, while two junctions in each cell type also had small insertions of 1–3 bp. The mean length of microhomology in CNVs from wild-type and *Xrcc4^−/−^* cells was 2.0 bp and 2.1 bp, respectively, and the median length for both was 2.0 bp. The lack of a shift toward longer microhomologies in the absence of Xrcc4 strongly argues against a shift from utilization of canonical NHEJ toward alt-EJ in Xrcc4-deficient cells, and therefore that these junctions were not formed by Xrcc4-dependent DSB repair, even in wild-type cells.

**Table 2:** APH-induced *de novo* CNV breakpoint junctions.

Clone	Genotype	[APH]	CNV type	Chr	Left breakpoint (hg18)	Right breakpoint (hg18)	# bp homology at junction	Homologous bases	Inserted bases
X6-5	Xrcc4−/−	0.6	Deletion	1	68,450,738	68,966,178	1	A	-
X6-10	Xrcc4−/−	0.6	Deletion	2	67,650,700	67,818,163	2	TT	-
X6-5	Xrcc4−/−	0.6	Deletion	2	140,718,860	141,009,164	3	CAA	-
WT6-31	WT	0.6	Deletion	2	162,172,198	162,232,243	0	-	-
X6-11	Xrcc4−/−	0.6	Deletion	3	156,164,457	156,204,161	2	TT	-
WT6-33	WT	0.6	Complex	4	153,025,685	153,222,760	1	G	-
WT6-33	WT	0.6	Complex	4	153,025,694	153,222,677	0	-	TG
WT6-33	WT	0.6	Complex	4	153,222,732	153,025,763	2	TG	-
WT6-33	WT	0.6	Complex	4	153,222,767	153,222,638	4	AACA	-
WT6-3	WT	0.6	Complex	5	5,038,414	5,140,617	3	CAT	-
WT6-3	WT	0.6	Complex	5	5,141,891	5,167,320	4	GGTA	-
X6-10	Xrcc4−/−	0.6	Deletion	5	132,199,490	132,455,826	0	-	-
WT6-3	WT	0.6	Deletion	5	132,226,382	132,474,192	0	-	A
WT6-1	WT	0.6	Deletion	5	132,476,605	132,681,396	1	A	-
X6-37	Xrcc4−/−	0.6	Deletion	5	132,488,580	132,582,758	2	CA	-
WT6-33	WT	0.6	Deletion	5	132,621,299	132,650,279	1	T	-
X6-11	Xrcc4−/−	0.6	Complex	5	132,624,159	132,850,747	3	CAG	-
X6-11	Xrcc4−/−	0.6	Complex	5	132,850,831	132,844,066	2	AC	-
X6-40	Xrcc4−/−	0.6	Deletion	6	77,628,529	77,694,182	0	-	ATA
X6-6	Xrcc4−/−	0.6	Complex	8	30,060,662	30,079,853	3	TCA	-
X6-6	Xrcc4−/−	0.6	Complex	8	30,080,507	30,102,797	4	TCTG	-
X6-6	Xrcc4−/−	0.6	Complex	8	30,103,083	30,107,692	5	AGCTC	-
WT6-1	WT	0.6	Deletion	9	28,994,436	29,024,833	2	GT	-
X6-7	Xrcc4−/−	0.6	Duplication	9	69,489,187	68,994,934	2	CA	-
WT6-4	WT	0.6	Deletion	11	25,829,101	25,853,015	5	T(T/C)CTGC	-
X6-5	Xrcc4−/−	0.6	Deletion	11	47,093,534	47,349,312	2	TC	-
X6-20	Xrcc4−/−	0.6	Deletion	12	38,642,366	38,735,475	2	TT	-
WT6-32	WT	0.6	Deletion	12	80,456,477	80,489,599	3	GAG	-
WT6-14	WT	0.6	Deletion	14	23,303,767	23,354,878	0	-	-
X6-13	Xrcc4−/−	0.6	Deletion	14	30,009,560	30,339,884	4	ACTA	-
X6-35	Xrcc4−/−	0.6	Complex	16	49,786,986	49,809,344	2	GG	-
X6-35	Xrcc4−/−	0.6	Complex	16	49,809,524	49,805,150	3	GCA	-
X6-4	Xrcc4−/−	0.6	Deletion	16	66,978,951	67,305,221	1	C	-
WT6-14	WT	0.6	Deletion	16	97,254,626	97,308,282	1	C	-
X6-4	Xrcc4−/−	0.6	Deletion	18	72,162,955	72,298,910	0	-	CA
X6-9	Xrcc4−/−	0.6	Deletion	X	40,273,857	40,335,846	1	C	-
WT6-4	WT	0.6	Deletion	X	58,904,820	59,057,091	3	AGG	-
WT6-43	WT	0.6	Deletion	X	80,613,171	80,738,690	0	-	-
X6-6	Xrcc4−/−	0.6	Deletion	X	84,741,276	84,811,809	0	-	-
X6-19	Xrcc4−/−	0.6	Deletion	X	110,651,646	110,711,246	2	TT	-
X6-4	Xrcc4−/−	0.6	Deletion	X	126,700,368	126,786,727	0	-	-

Similarly, none of the sequenced junctions had long stretches of homology that would suggest a shift toward HR in NHEJ-deficient cells. To explore this further, we examined the breakpoint regions of unsequenced deletions *in silico* to determine if *Xrcc4^−/−^* cells had an increased breakpoint frequency near segmental duplications that might suggest formation by HR. For each CNV, 10 kb windows of sequence from the left and right breakpoint regions were compared to each other, scoring instances of sequence identity >90% along a stretch of sequence at least 1000 bp. Such large sequence homologies were associated with only 3.5% and 4.0% of CNVs in wild-type and *Xrcc4^−/−^* cells, respectively (p = 1.0), reinforcing that there is no apparent increase in sequence homology at breakpoint regions in Xrcc4-deficient cells.

### Similar complex CNVs occur in wild-type and *Xrcc4^−/−^* cells

**Figure 5:**
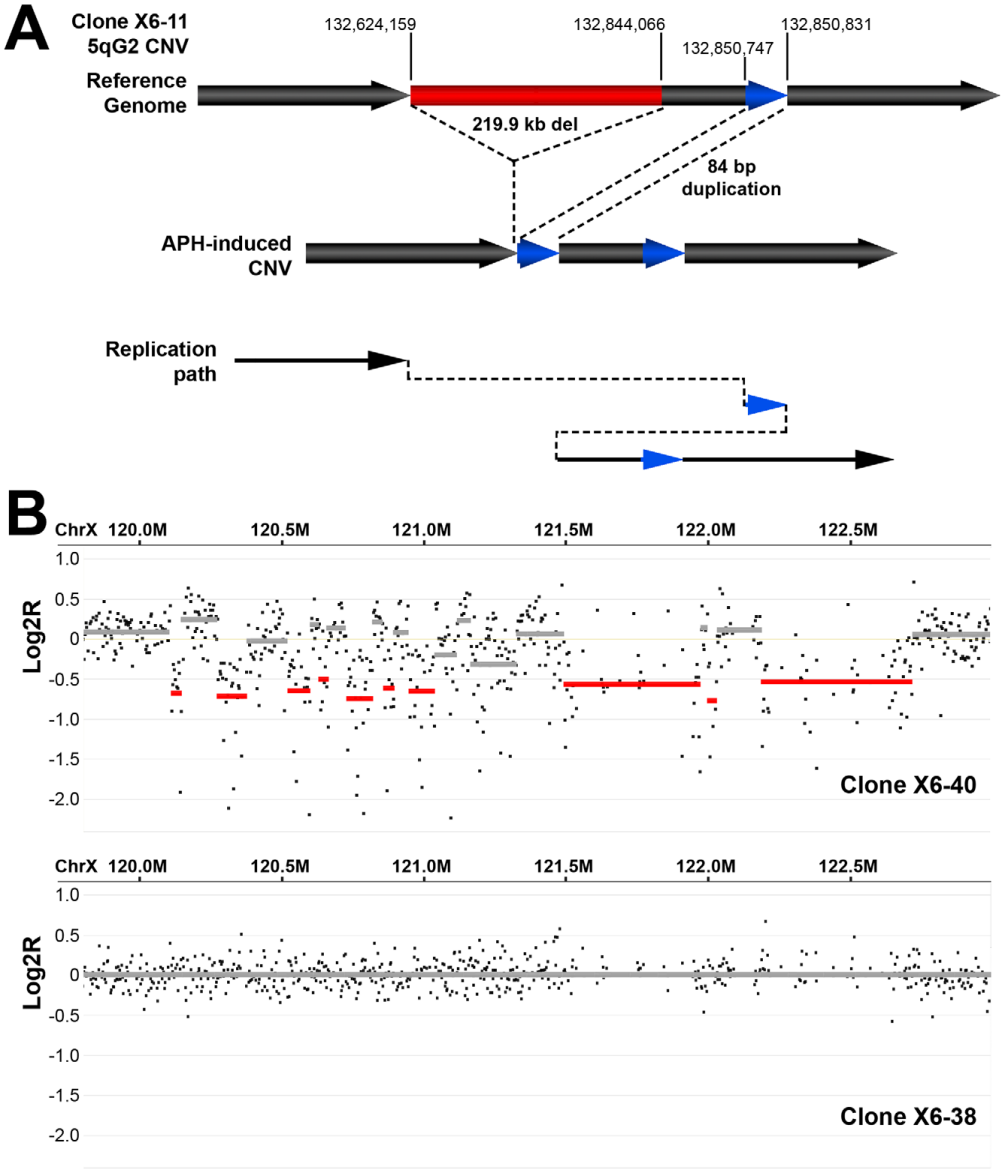
Example of complex APH-induced *de novo* CNVs in mouse ES cells. (A) A complex CNV with two junctions at 5G2 in APH-treated *Xrcc4^−/−^* clone X6-11. Based on aCGH data, this CNV was called as a deletion, but sequencing of the breakpoint junctions revealed that this CNV was complex, containing a 219.9 kb deletion (red), as well as a duplication-insertion of 84 bp (blue) at the deletion boundary. (B) aCGH data demonstrating a region of complex CNV in APH-treated *Xrcc4^−/−^* clone X6-40 at XE3 containing 10 or more discrete deletions across a ∼2.5 Mb region. Data from the same genomic interval in a control clone (X6-38) is shown for comparison.

Thirteen of the sequenced breakpoint junctions were from five complex CNVs that contained two to four breakpoint junctions each. These CNVs recapitulate the type of complex events seen in human fibroblasts [42], [43] and *in vivo* [19], [38], [39], [40]. Two of these complex CNVs were found in wild-type cells and three were from *Xrcc4^−/−^* clones, again suggesting no Xrcc4-dependent structural difference. These complex CNVs were initially scored as simple deletions based on aCGH data, but sequencing revealed the presence of small retained sequences, as well as duplications and inversions that were below the resolution limit of the array (Figure 5A, Figure S5). In addition, *Xrcc4^−/−^* clone X6-40 contained a 2.5 Mb region of chromosome XE3 containing at least 10 discrete deletions (Figure 5B). This CNV is similar to the recently-described chromothripsis class of structural alterations [45]. Finally, we note that we successfully sequenced CNV breakpoint junctions in only 41 out of 60 attempts (68%). The CNVs for which breakpoint cloning failed likely include some junctions with complex structures that are difficult to amplify. Accordingly, we expect that our six complex CNVs are an underrepresentation of the actual incidence of such events.

## Discussion

The experiments reported here demonstrate that APH-induced replication stress creates *de novo* CNVs in mouse ES cells that mimic *in vivo* nonrecurrent CNVs in the same manner as in human fibroblasts, and that these and spontaneous CNVs arise independently of Xrcc4-dependent NHEJ. Neither the frequency nor any observable feature of location or structure of APH-induced CNVs was affected by Xrcc4 loss. Almost all *de novo* CNVs in both wild-type and *Xrcc4^−/−^* cells had breakpoint regions devoid of the extended sequence homology needed to drive HR. Detailed characterization of individual breakpoint junctions confirmed that the CNVs arose via a non-homologous mechanism characterized by blunt ends, short microhomologies or short insertions, regardless of Xrcc4 status. These results eliminate canonical NHEJ as a primary mechanism for *de novo* CNV formation in our cell system. Moreover, the identification of complex, chromothripsis-like events in *Xrcc4^−/−^* cells suggest this rearrangement can occur in the absence of the NHEJ pathway. Instead, the findings together implicate alt-NHEJ and/or replication template switching as the principal mediator(s) of nonhomologous junction formation.

**Figure 6:**
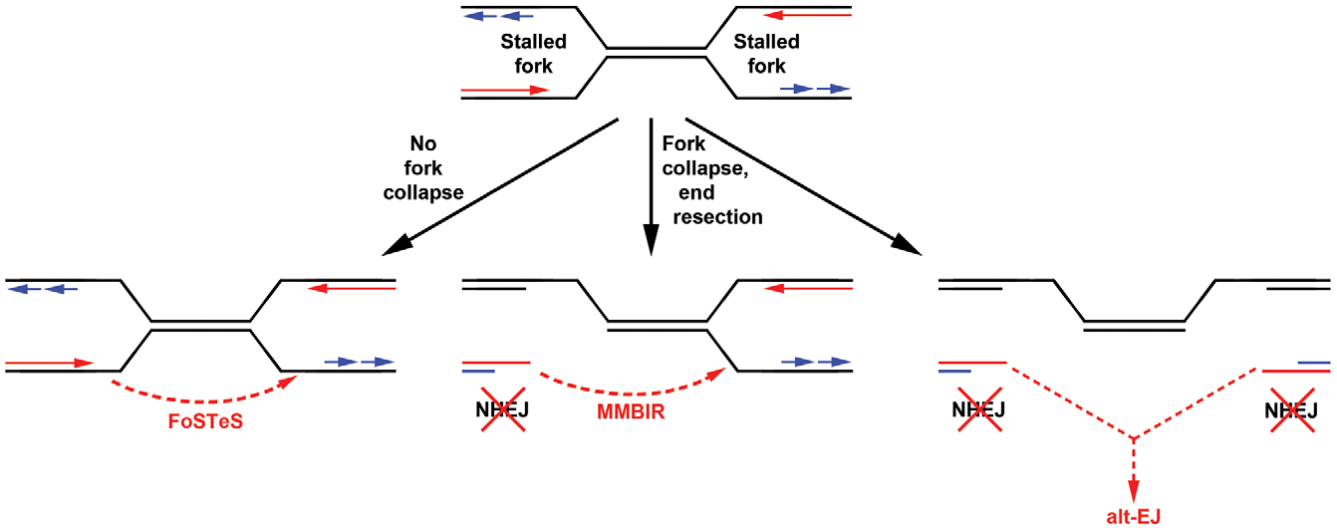
Models for replication-dependent, Xrcc4-independent CNV formation. The induction of CNVs by replication stress strongly implicates stalled replication as a key intermediate (top). Template switching without fork collapse might directly create CNVs without DSB formation (left). Alternatively, fork collapse and end processing might lead to iterative template copying prior to final stable resolution of single-ended DSBs by either maturation of a one DSB end into a replication fork (MMBIR, middle) or joining of two distant DSBs by alt-EJ (right). In neither case are the single-ended DSBs good substrates for NHEJ. Results here establish that Xrcc4-dependent NHEJ is neither required for, nor suppresses, CNV formation via these inferred intermediates.

In many ways, the results of this CNV study are similar to observations made using a two-DSB translocation model system [35], [46], [47]. Jasin and colleagues have shown that alt-EJ rather than canonical NHEJ likely acts in the formation of translocations following DSB induction, even when a functional NHEJ pathway is present. Similar to results here, they found that loss of Xrcc4 does not change the nature of translocation breakpoint junctions, which, like those seen at APH-induced CNVs, are typically characterized by 0–4 bp of microhomology. In addition, translocation junctions were sometimes complex, containing multiple insertions that were duplicated from sequences that could be as much as 4 Mb away from the initiating DSB, suggesting that iterative DNA synthesis occurred prior to joint resolution. These similar results could indicate that alt-EJ is playing a role in the CNVs induced in our system. However, a key difference is that loss of Xrcc4 and NHEJ increased DSB-induced translocations 5-fold [35], whereas loss of Xrcc4 did not significantly alter the frequency of CNV induction. This lack of CNV suppression might suggest that the precursor lesion for CNVs is distinct from the translocation model in that it can be processed by alt-EJ but not NHEJ. A powerful way to rationalize this would be creation of CNVs by joining of two single-ended DSBs formed at different collapsed replication forks (Figure 6). Individually, such replication-dependent DSBs are not substrates for local NHEJ and might, by analogy to the translocation model, be processed primarily by alt-EJ when joined at a distance. Alternatively, the lack of CNV suppression by NHEJ might suggest that DSB end joining is not an important contributor to CNV formation. Replication template switching, including FoSTeS and MMBIR [23], [37], are strong alternative models that are entirely consistent with all results here, including the lack of dependence of both CNV structure and frequency on Xrcc4 (Figure 6).

Importantly, alt-EJ and template switching models of CNV formation are not mutually exclusive, and indeed might be considered more similar than different (Figure 6). The demonstration that CNV formation increases after replication stress in mouse as well as human cells strongly supports both replication-dependent alt-EJ and template switching mechanisms [42], , since the partial inhibition of replication fork progression leads to increased frequencies of fork stalling and collapse to single-ended DSBs. Moreover, both replication-dependent alt-EJ and MMBIR invoke a processed single-ended DSB intermediate that could execute the sometimes multiple iterative template copying events that underlie the species- and cell type-independent occurrence of complex CNVs. What distinguishes replication-dependent alt-EJ and template switching is simply the mode of final joint resolution, which for alt-EJ is ligation to a second DSB end but for template switching is stabilization of the ultimate template copying event into a mature replication fork (Figure 6). Evidence that the latter event occurs is the induction of tandem duplication CNVs by replication stress, since duplications are easily explained by a template switch upstream of the DSB end. In total, though, both alt-EJ and maturation of template copying events might be used to resolve one-ended replication DSBs in different CNV events.

The results from this study also relate to the nature of chromothripsis events and CNV hotspots. Chromothripsis is a recently-described, catastrophic chromosome rearrangement seen in 2–3% of cancers [45]. It is also seen as a constitutional event in humans, suggesting that it is not specific to aberrant DNA repair pathways seen in cancer [48], [49]. These complex rearrangements are thought to occur as a single catastrophic event, rather than accumulating over time [50], [51]. The detection of a chromothripsis-like event in a *Xrcc4^−/−^* clone suggests that these catastrophic rearrangements can occur via an NHEJ-independent pathway, in contrast to chromosome shattering followed by religation via NHEJ [50]. Such catastrophic events can be explained under a unifying template switching model of CNV formation, in which the same basic replication stalling mechanism can give rise to simple as well as highly complex CNVs.

As with human cells, *de novo* CNVs in mouse cells are distributed across the genome, but include hotspots. Only some of these hotspots correspond to the syntenic regions of hotspots in human fibroblasts, indicating that hotspot conservation can vary between cell types and perhaps species. As seen in human fibroblasts, some CNV hotspots in mouse ES cells correspond with molecularly-characterized common fragile sites or in cytogenetic bands that contain fragile sites [42], [52], [53]. This correlation is not perfect, and several hotspots do not correspond to any known fragile site region. However, fragile sites have largely been characterized in primary lymphocytes and lymphoblastoid cell lines, and it is known that different cell types can have altered fragile site expression [53]. It is therefore possible that additional hotspots seen in mouse ES cells and human fibroblasts correspond to fragile sites that are preferentially expressed in those cells.

The observation that hotspot CNVs are almost all deletions and tend to be almost twice as large as non-hotspots, coupled with the possible fragile site connection, raises interesting possibilities for their formation. APH and hydroxyurea are known to activate the firing of dormant replication origins in *Xenopus* extracts and mammalian cells [54], [55]. It has been shown that common fragile sites can occur in regions with a paucity of activated origins after replication stress, resulting in delayed and incomplete replication [56], [57], [58]. The lower active origin density results in a larger mean distance between active replication forks in these regions. If a replication-based mechanism involving either alt-EJ or template-switching between forks is responsible for CNVs, the greater fork spacing in regions with low active origin density would result in a larger CNV size, and large unreplicated regions that persist beyond S-phase could favor the formation of deletions over duplications, as observed in our experiments. While origin paucity is characteristic of fragile sites, incomplete activation of primary or dormant origins could also play a role in CNV formation at non-hotspots.

In summary, the experiments described here demonstrate that canonical, Xrcc4-dependent NHEJ is not involved in CNV formation in somatic cells cultured *in vitro*. Evidence from breakpoint junction structures further demonstrates that the CNVs did not form via HR. Instead, the data implicate a replication-dependent alt-EJ and/or template switching mechanism. Because of the strong similarity of the observed CNVs to the major classes of nonrecurrent normal and pathogenic CNVs seen in humans, we argue that these conclusions are generalizable to most *de novo*, nonrecurrent CNV formation in both germline and somatic human cell lineages, with the simple difference that event rates are higher in our model system because replication is exogenously stressed. Although not mutually exclusive, important features distinguish the remaining alt-EJ and template switching mechanisms, specifically the manner in which the strands are stably resolved. Also enigmatic is precisely which DNA intermediate is the substrate for template switching and which proteins are involved in executing the transfer when little or no microhomology is present. Major efforts moving forward should thus be to delineate the precise strand intermediates and protein mechanisms involved in mediating nonrecurrent CNV formation.

## Materials and Methods

### Generation of cell clones containing replication stress-induced CNVs

All experiments were performed with two isogenic male mouse ES cell lines. The first (TC1) was wild-type, while the second (*Xrcc4^−/−^*) was homozygous for a targeted inactivation of *Xrcc4* [59]. Genomic DNA was prepared from cells using the Blood & Cell Culture DNA Mini Kit (Qiagen). ES Cells were grown irradiated fibroblast feeder cells in DMEM media supplemented with 15% FBS, 20 mM HEPES, and 1 mM sodium pyruvate. To create replication stress-induced CNVs, cells were treated with 0.6 µM APH. In three independent experiments, cells were treated for 72 hours followed by a 24 hour recovery period before plating at low density for single-cell clones. Cells were plated at a density of 100–500 cells per 100-mm culture dish and individual clones isolated with a pipette tip after 7–10 days.

### aCGH

CNVs were detected using Nimblegen whole genome arrays containing 720,000 (720K) unique sequence oligonucleotides (Roche Applied Science). Arrays were prepared according to the manufacturer's protocol. Arrays were scanned on an Axon 4000B scanner (Molecular Devices) with GenePix software at 532 and 635 wavelengths. Data extraction, normalization, and visualization were achieved by using manufacturer-provided software (NimbleScan and Signal-Map). Arrays were analyzed for copy number differences using SegMNT, part of Nimblegen's NimbleScan software package, as well as our software platform, VAMP, as previously described [19]. All clones were analyzed using the appropriate mixed parental cell population as the normalization reference. This approach routinely detects CNVs larger than 20 kb and can detect CNVs as small as a ∼1 kb, depending on probe placement.

### CNV breakpoint junctions

CNV breakpoint junctions were amplified using the Expand Long Template PCR System (Roche Applied Science). For deletions, PCR primer pairs were generated that flanked deletion breakpoints, whereas for duplications, primers were designed within the duplicated region, directed outward, as described previously [43]. PCR amplification generated a product that spanned the breakpoint junction. All products were then subjected to standard Sanger sequencing. The resulting sequence was compared to the reference genome (build mm9) to identify the breakpoint junctions.

### Statistical methods

CNVs in our model system are relatively rare events and therefore the numbers of CNVs per clone are expected to fit a Poisson distribution determined by the mean frequency of CNVs in all clones. Therefore, p values of treated vs. untreated samples were determined using the one-sided E-test of Krishnamoorthy and Thomson for comparing two Poisson mean rates [60].

To determine whether the observed clustering of CNVs within genome regions was non-random, we performed the Monte Carlo simulation summarized in Table S1, as previously described for human cells [42]. A simulation of 10,000 iterations was performed on the combined wild-type and Xrcc4^−/−^ CNV sets. Regions with 5 or more overlapping CNVs were very rarely observed by random placement (p<0.01, Table S1) and were therefore scored as CNV hotspots in mouse ES cells. These hotspot regions are highlighted by shading in Table S2.

## Supporting Information

Figure S1 Confirmation of *Xrcc4−/−* mutant mouse ES cell line. (A) PCR confirmation of mutant *Xrcc4* allele with deletion of exon 3 [52]. PCR primers: XFor1 GCTGAGTACTTAGATTTGAGTAC; XRev1 ACCTGGGTGACCCTTACACG. (B) IR sensitivity of *Xrcc4−/−* ES cells. Wild-type and *Xrcc4−/−* cells were irradiated with indicated doses of X irradiation, cultured for 7 days, and surviving colonies were stained and counted. IR sensitivity is expressed as the percentages of surviving colonies over unirradiated controls.
(TIF)

Figure S2 Examples of APH-induced CNVs showing Nimblegen aCGH intensity data (log2R). Each dot represents a single probe on the array. (A) A 107.0 kb deletion at 8E1 in clone X6-21 is easily detected by a reduction in the log2R intensity. (B) A 486.6 kb duplication at 9C–D in clone X6-7 can be identified by an increase in the log2R values.
(TIF)

Figure S3 Box and whisker plot illustrating APH-induced CNV formation in wild-type (“WT”, blue) and *Xrcc4−/−* (red) cells, in each of three experiments. It is evident that wild-type cells from Experiment 1 formed unusually low numbers of *de novo* CNVs compared to all other experimental groups. As a result, when data are combined, there is an apparent increase in CNV formation in *Xrcc4−/−* cells (Figure 1A).
(TIF)

Figure S4 CNV coverage at all hotspots in mouse ES cells. The x-axis shows the position along the chromosome, while the y-axis indicates that fraction of hotspot CNVs that crossed a particular 10 kb genomic window.
(TIF)

Figure S5 Demonstration of complex CNV rearrangements in wild-type and *Xrcc4−/−* cells. Each of these CNVs was called as a deletion based on aCGH data. Breakpoint junction sequencing revealed small duplications (blue), interrupted deletions (red), and inversions (gray).
(TIF)

Table S1 Monte Carlo simulation to identify CNV hotspots.
(DOCX)

Table S2 List of *de novo* CNVs.
(XLSX)
