# Supplementary figures and images for: De Novo CNV Formation in Mouse Embryonic Stem Cells Occurs in the Absence of Xrcc4-Dependent Nonhomologous End Joining
Source: PLoS Genet. 2012 Sep 20;8(9):e1002981. doi: 10.1371/journal.pgen.1002981 (PMC3447954; doi:10.1371/journal.pgen.1002981)

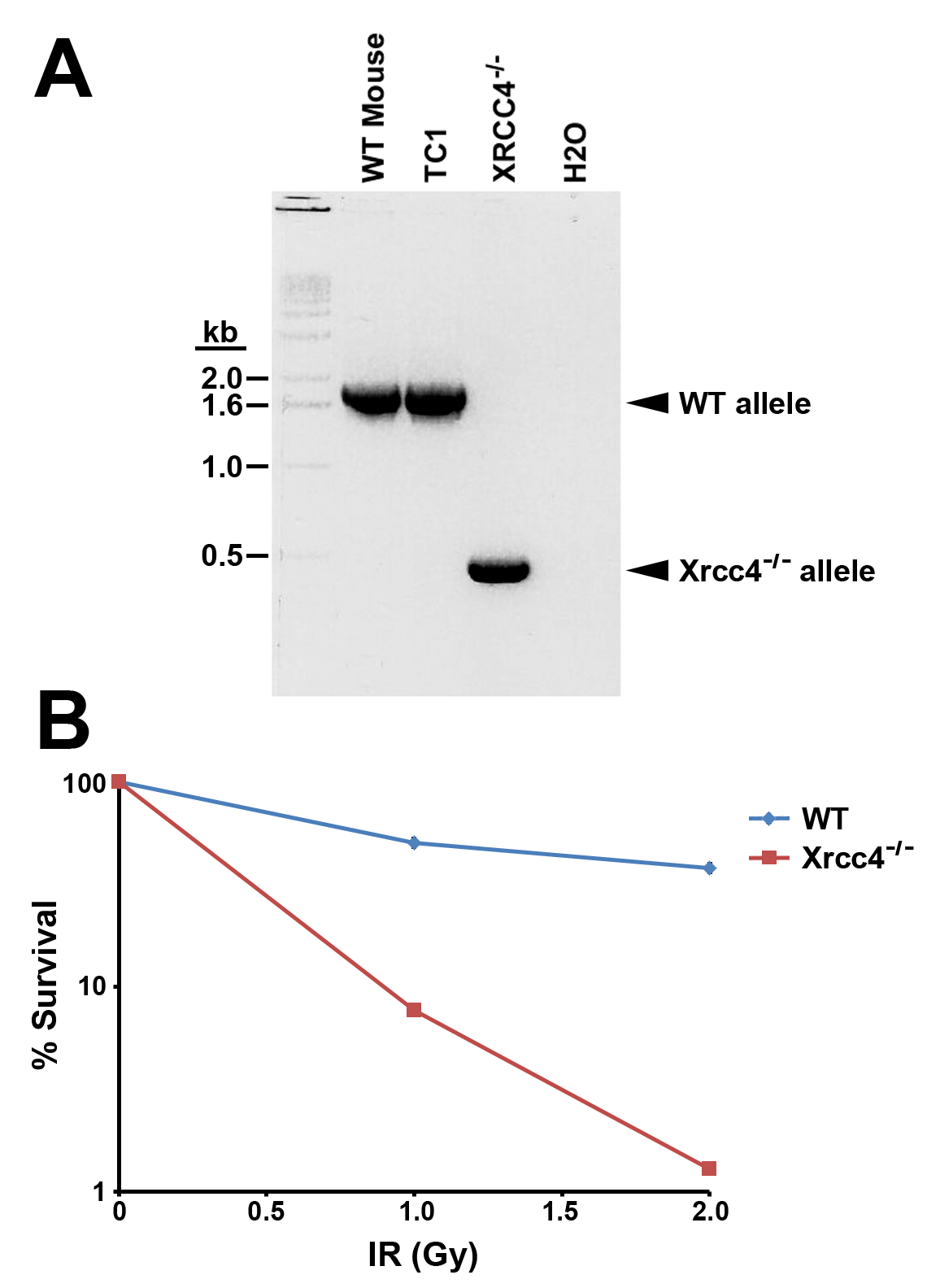

Supplement: Figure S1 — Confirmation of Xrcc4−/− mutant mouse ES cell line. (A) PCR confirmation of mutant Xrcc4 allele with deletion of exon 3 [52]. PCR primers: XFor1 GCTGAGTACTTAGATTTGAGTAC; XRev1 ACCTGGGTGACCCTTACACG. (B) IR sensitivity of Xrcc4−/− ES cells. Wild-type and Xrcc4−/− cells were irradiated with indicated doses of X irradiation, cultured for 7 days, and surviving colonies were stained and counted. IR sensitivity is expressed as the percentages of surviving colonies over unirradiated controls. (TIF) [file pgen.1002981.s001.tif]

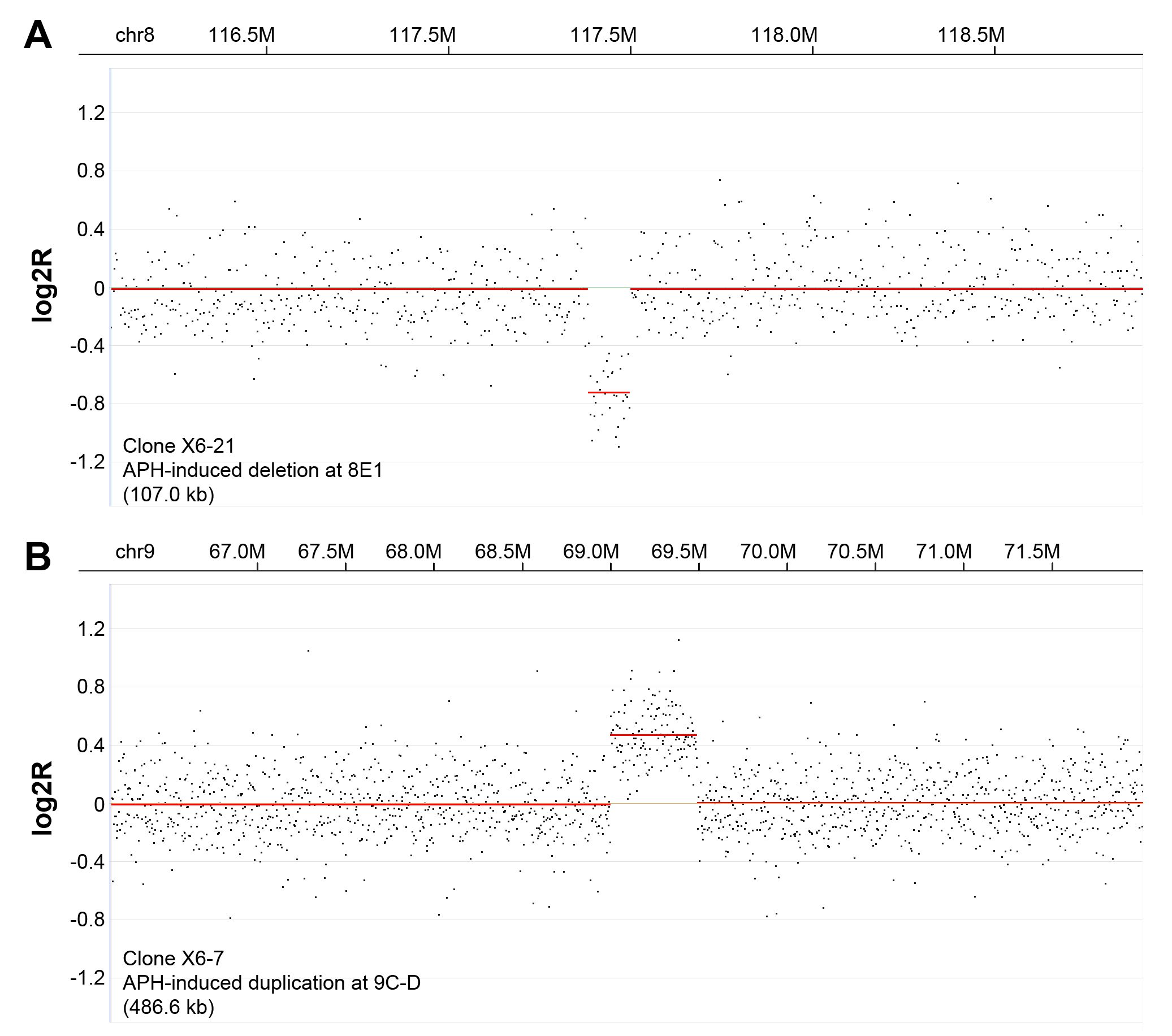

Supplement: Figure S2 — Examples of APH-induced CNVs showing Nimblegen aCGH intensity data (log2R). Each dot represents a single probe on the array. (A) A 107.0 kb deletion at 8E1 in clone X6-21 is easily detected by a reduction in the log2R intensity. (B) A 486.6 kb duplication at 9C–D in clone X6-7 can be identified by an increase in the log2R values. (TIF) [file pgen.1002981.s002.tif]

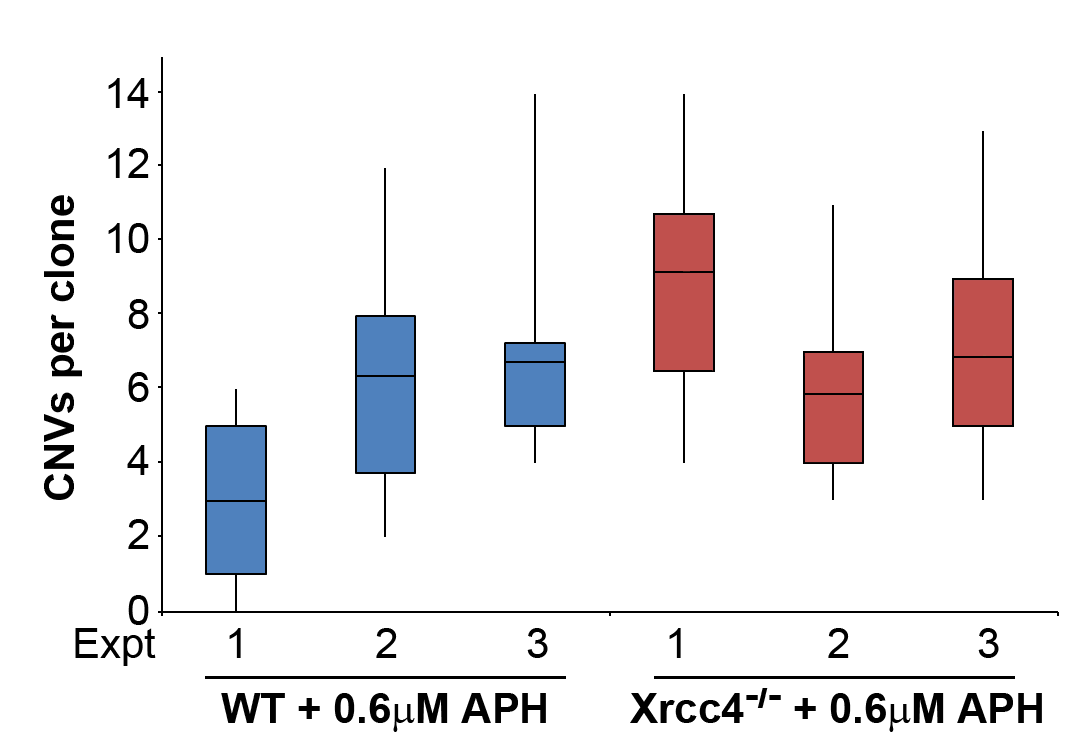

Supplement: Figure S3 — Box and whisker plot illustrating APH-induced CNV formation in wild-type (“WT”, blue) and Xrcc4−/− (red) cells, in each of three experiments. It is evident that wild-type cells from Experiment 1 formed unusually low numbers of de novo CNVs compared to all other experimental groups. As a result, when data are combined, there is an apparent increase in CNV formation in Xrcc4−/− cells (Figure 1A). (TIF) [file pgen.1002981.s003.tif]

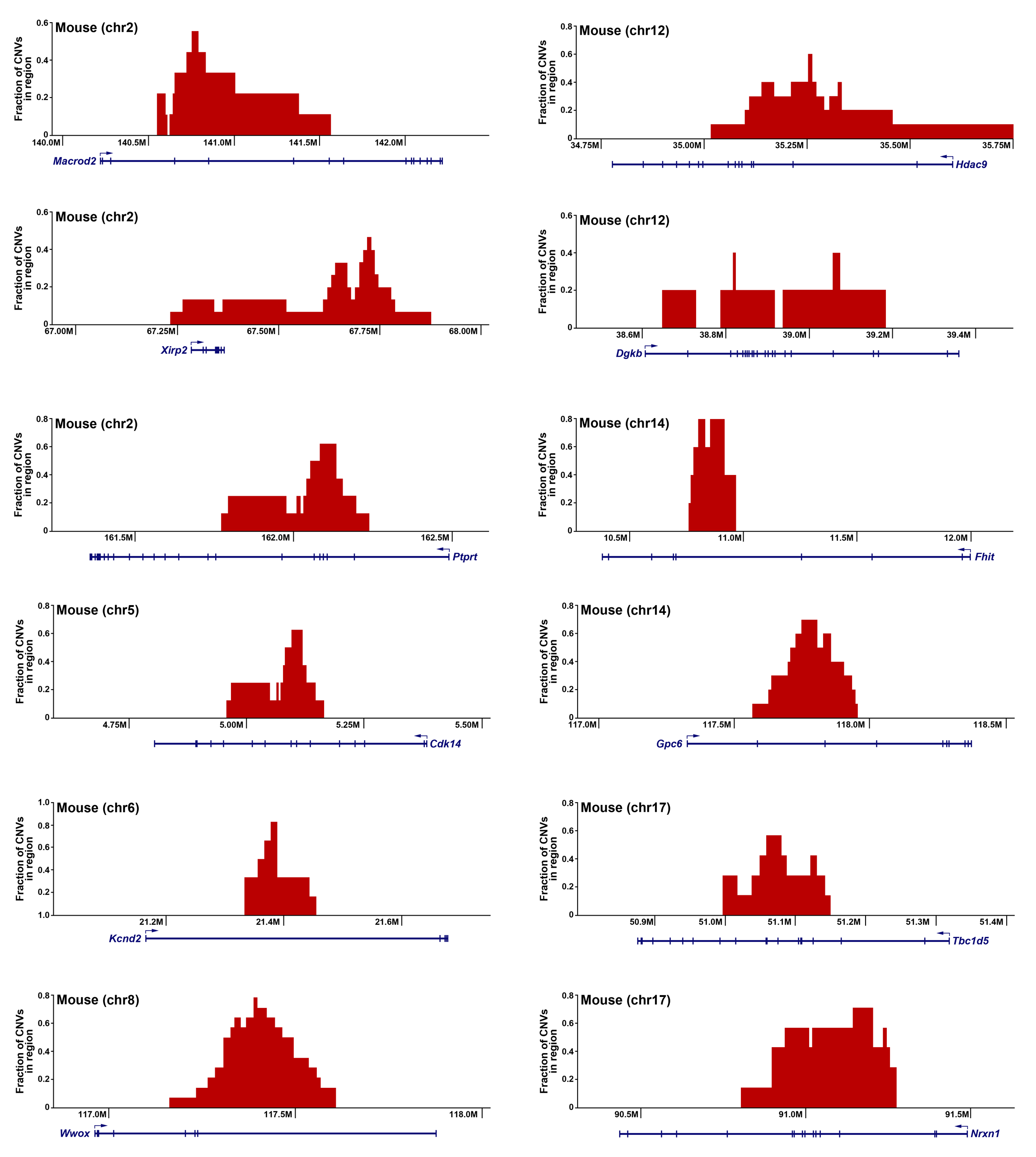

Supplement: Figure S4 — CNV coverage at all hotspots in mouse ES cells. The x-axis shows the position along the chromosome, while the y-axis indicates that fraction of hotspot CNVs that crossed a particular 10 kb genomic window. (TIF) [file pgen.1002981.s004.tif]

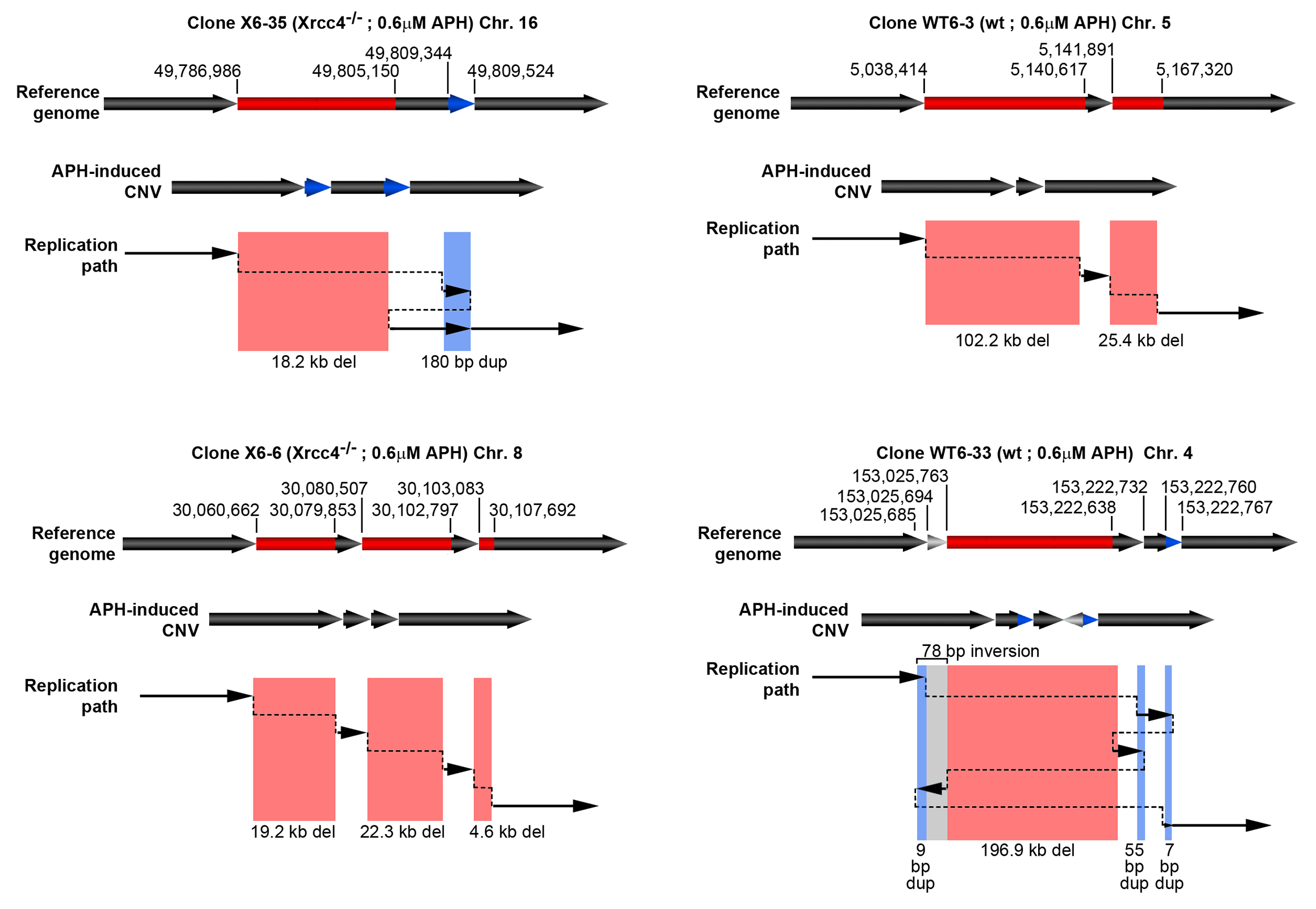

Supplement: Figure S5 — Demonstration of complex CNV rearrangements in wild-type and Xrcc4−/− cells. Each of these CNVs was called as a deletion based on aCGH data. Breakpoint junction sequencing revealed small duplications (blue), interrupted deletions (red), and inversions (gray). (TIF) [file pgen.1002981.s005.tif]
