# Supplementary material for: De Novo CNV Formation in Mouse Embryonic Stem Cells Occurs in the Absence of Xrcc4-Dependent Nonhomologous End Joining
Source: PLoS Genet. 2012 Sep 20;8(9):e1002981. doi: 10.1371/journal.pgen.1002981 (PMC3447954; doi:10.1371/journal.pgen.1002981)
Supplement: Table S1 — Monte Carlo simulation to identify CNV hotspots. (DOCX) [file pgen.1002981.s006.docx]

| **Table S1. Monte Carlo simulation to identify CNV hotspots.** | | | | | | |
| --- | --- | --- | --- | --- | --- | --- |
|  | **Observed CNVs** | | **Simulation Mean** | |  |  |
| **CNVs in region** | **Regions** | **CNVs** | **Regions** | **CNVs** | **p(>0)** | **p(obs)** |
| 1 | 151 | 151 | 330 | 330 | 1.0 | 1.0 |
| 2 | 25 | 50 | 18 | 35 | 1.0 | 0.06 |
| 3 | 8 | 24 | 1.1 | 3.2 | 0.7 | 0.00002 |
| 4 | 3 | 12 | 0.071 | 0.29 | 0.07 | 0.0000575 |
| 5 | 3 | 15 | 0.0038 | 0.02 | 0.0038 | 0.0000 |
| 6 | 1 | 6 | 0.0003 | 0.0018 | 0.0003 | 0.0003 |
| 7 | 2 | 14 | 0 | 0 | <0.0001 | <0.0001 |
| 8 | 1 | 8 | 0 | 0 | <0.0001 | <0.0001 |
| 9 | 2 | 18 | 0 | 0 | <0.0001 | <0.0001 |
| 10 | 1 | 10 | 0 | 0 | <0.0001 | <0.0001 |
| 14 | 1 | 14 | 0 | 0 | <0.0001 | <0.0001 |
| 15 | 1 | 15 | 0 | 0 | <0.0001 | <0.0001 |
| 32 | 1 | 32 | 0 | 0 | <0.0001 | <0.0001 |

Details in main text (*Materials and Methods*)
